# Innovation at the Intersection: Emerging Translational Research in Neurology and Psychiatry

**DOI:** 10.3390/cells13100790

**Published:** 2024-05-07

**Authors:** Masaru Tanaka, Simone Battaglia, Lydia Giménez-Llort, Chong Chen, Piril Hepsomali, Alessio Avenanti, László Vécsei

**Affiliations:** 1 HUN-REN-SZTE Neuroscience Research Group, Hungarian Research Network, University of Szeged (HUN-REN-SZTE), Danube Neuroscience Research Laboratory, Tisza Lajos krt. 113, H-6725 Szeged, Hungary; vecsei.laszlo@med-u-szeged.hu; 2 Center for Studies and Research in Cognitive Neuroscience, Department of Psychology “Renzo Canestrari”, Cesena Campus, Alma Mater Studiorum Università di Bologna, 47521 Cesena, Italy; alessio.avenanti@unibo.it; 3 Department of Psychology, University of Turin, 10124 Turin, Italy; 4 Institut de Neurociències, Universitat Autònoma de Barcelona, Cerdanyola del Vallès, 08193 Barcelona, Spain; lidia.gimenez@uab.cat; 5 Department of Psychiatry & Forensic Medicine, Faculty of Medicine, Campus Bellaterra, Universitat Autònoma de Barcelona, Cerdanyola del Vallès, 08193 Barcelona, Spain; 6 Division of Neuropsychiatry, Department of Neuroscience, Yamaguchi University Graduate School of Medicine, Yamaguchi 755-8505, Japan; cchen@yamaguchi-u.ac.jp; 7 School of Psychology and Clinical Language Sciences, University of Reading, Reading RG6 6ET, UK; p.hepsomali@reading.ac.uk; 8 Neuropsychology and Cognitive Neuroscience Research Center (CINPSI Neurocog), Universidad Católica del Maule, Talca 3460000, Chile; 9 Department of Neurology, Albert Szent-Györgyi Medical School, University of Szeged, Semmelweis u. 6, H-6725 Szeged, Hungary

## 1. Introduction

Translational research in neurological and psychiatric diseases is a rapidly advancing field that promises to redefine our approach to these complex conditions [1,2,3,4,5,6,7,8,9]. The Topic “Emerging Translational Research in Neurological and Psychiatric Diseases” is a testament to this evolution, showcasing 22 pioneering papers that span a diverse range of topics and methodologies. This Editorial aims to provide a cohesive overview of the collection, emphasizing the synergy between various research domains and the translational potential of the findings presented. These papers fall into the following subtopics: Advancements in Neuroimaging and Neurostimulation, Insights into Neurodegenerative and Neuroinflammatory Disorders, and Exploring Neuropsychiatric Disorders and Treatments.

The first subtopic opens with a series of papers that explore the frontiers of neuroimaging and neurostimulation. These studies not only enhance our understanding of brain morphology and function, but also introduce groundbreaking techniques for modulating brain activity [10,11,12,13,14,15,16]. Non-invasive brain stimulation techniques, including transcranial magnetic stimulation (TMS), transcranial direct current stimulation, neurofeedback, and deep brain stimulation (DBS), are frequently used to influence neural activity in specific brain regions, providing therapeutic benefits for conditions such as depression and anxiety [17,18,19,20,21,22,23]. The implications for diagnosis and therapy are profound, offering new pathways for addressing neurological and psychiatric disorders that were once considered intractable [24,25,26,27,28,29,30,31,32].

A central theme of the second subtopic is the in-depth examination of neurodegenerative and neuroinflammatory disorders such as Alzheimer’s disease (AD); this includes exploring strategies for neural regeneration and repair. Neuroplasticity, the brain’s ability to reorganize itself by forming new neural connections, is particularly relevant in neurodegenerative diseases [33,34,35,36,37,38]. Interventions targeting neural plasticity aim to slow cognitive decline by promoting adaptive changes in the brain’s structure through cognitive training, physical exercise, or environmental enrichment [39,40,41,42,43,44,45]. The papers delve into the pathophysiology of diseases such as AD, highlighting the latest strategies for neural regeneration and repair. The inclusion of research on rare and genetic neurological disorders broadens the scope, providing insights into the genetic factors that influence neurology and the potential for personalized medicine [46,47,48,49,50,51].

The third subtopic also casts a spotlight on neuropsychiatric disorders, offering a nuanced look at conditions such as depression and their treatments [52,53,54,55,56,57]. Topics include the role of antidepressants, the importance of neural oscillations in cognitive functions, and the interplay between neurotrophic factors and genetic variants. These papers underscore the complexity of mental health and the multifaceted nature of effective treatment strategies. Each paper in this collection contributes to a mosaic of knowledge that enhances our understanding of the brain and its disorders. Employing both qualitative and quantitative methods, the researchers provide a rich tapestry of data that not only reflects the current state of the field, but also lays the groundwork for future innovations [58,59,60,61]. As we look ahead, the insights garnered from this collection will undoubtedly inform the next generation of translational research, ultimately leading to improved outcomes for patients worldwide.

## 2. Topic Articles

### 2.1. Neuroimaging and Neurostimulation Techniques

Neuroimaging and neurostimulation techniques encompass a diverse range of studies that explore the intricate workings of the brain and the potential therapeutic interventions for neurological conditions [10,11,18,27,62,63,64]. From a systematic review of brain morphometric changes throughout life to the nuanced effects of ultrasound on cortical activity, these articles shed light on the dynamic field of neuroimaging [12,20,22,28,29,65,66]. Furthermore, the authors explore the field of neurostimulation, specifically examining the potential therapeutic applications of arginine–vasopressin receptor antagonists in the treatment of stroke, the transformative capabilities of DBS in the context of neurodegenerative disorders, and the safety implications associated with transcranial electrical stimulation. Additionally, cutting-edge research on electroencephalography (EEG) biomarkers using machine learning underscores innovative approaches being undertaken to understand and predict outcomes in disorders of consciousness. Collectively, these studies represent the forefront of neuroscientific research, offering insights and raising questions about the future of brain health and therapy.

#### 2.1.1. Neuroimaging Techniques for Brain Morphometry and Function

One of the main challenges in neuroscience is understanding how the brain works in health and disease [67,68,69]. To achieve this goal, researchers use various methods to measure the structure and activity of the brain, such as magnetic resonance imaging (MRI), EEG, and positron emission tomography (PET). These methods provide information about the anatomy, function, metabolism, and connectivity of the brain regions involved in different cognitive processes, emotions, and behaviors. They are also used to diagnose and monitor various neurological and psychiatric disorders, such as stroke, AD, schizophrenia (SCZ), and depression [70,71,72,73,74]. In this subsection, we review two papers that explore the use of different neuroimaging techniques for measuring the structure and activity of the brain.

Statsenko et al. propose a protocol for the systematic review and meta-analysis of longitudinal changes in brain morphology across the lifespan, from infancy to old age, using MRI data from different cohorts and studies [75]. The authors aim to search for characteristic features of non-pathological development and degeneration in distinct brain structures and devise a precise descriptive model of brain morphometry in age groups. They expect to find age-related patterns of brain morphometry changes that can serve as normative references for clinical studies and diagnoses of neurodegenerative diseases. The authors also plan to explore the potential factors that may influence brain morphometry, such as sex, genetics, lifestyle, and environmental exposures. They hope that their study will provide a comprehensive overview of the current knowledge and gaps in the field of brain morphometry and aging, and stimulate further research on this topic (Table 1).

Di Gregorio et al. evaluated the accuracy of EEG biomarkers in predicting the clinical outcome of patients with disorders of consciousness following severe acquired brain injury, using a machine learning approach to analyze EEG signals recorded during resting state and auditory stimulation [76]. The authors enrolled 20 patients with disorders of consciousness and 20 healthy controls, and performed EEG recordings at baseline and after one month. They extracted several features from the EEG signals, such as spectral power, coherence, entropy, and complexity, and used a support vector machine classifier to discriminate between patients and controls, and between patients with different levels of consciousness. The authors found that the EEG features had high accuracy in classifying the patients and the controls, and were correlated with the clinical outcome of the patients. They concluded that EEG biomarkers serve as a reliable and objective tool for assessing the level of consciousness and prognosis of patients with disorders of consciousness.

#### 2.1.2. Neurostimulation Techniques for Brain Modulation and Therapy

Neurostimulation techniques are promising approaches for modulating the brain activity and function in various neurological and psychiatric conditions, such as stroke, Parkinson’s disease (PD), depression, and chronic pain [10,11,18,20,97,98,99,100]. They can also be used to enhance cognitive performance, learning, and memory in healthy individuals [101,102,103]. These techniques involve applying different types of stimuli, such as ultrasounds, electric currents, magnetic fields, or drugs, to specific brain regions or networks, in order to alter their excitability, connectivity, or plasticity [104,105,106,107,108,109,110,111]. In this subtopic, we review four papers that explore the use of different neurostimulation techniques for brain modulation and therapy.

The first paper investigates the effects of weak ultrasound on the rat motor cortex, and suggests that it can induce neuromodulation without causing tissue damage [77]. The authors used functional MRI (fMRI) and electrophysiological recordings to measure the changes in blood-oxygen-level-dependent (BOLD) signals and local field potentials (LFPs) in response to ultrasound stimulation. They found that ultrasound increased the BOLD signals and LFPs in the stimulated area, as well as in the contralateral motor cortex and the thalamus. They also observed that ultrasound enhanced the motor-evoked potentials elicited by TMS, indicating an increase in corticospinal excitability. The authors concluded that weak ultrasound can be used as a non-invasive and focal neurostimulation technique for modulating the motor cortex.

The second paper reviews the role of arginine–vasopressin (AVP) in stroke, and proposes that its type 1 receptor antagonists (V1RAs) can be used as a novel neuroprotective strategy [78]. The authors summarized the evidence that AVP is involved in the pathophysiology of stroke, such as increasing blood pressure, promoting inflammation, inducing cerebral edema, and impairing cerebral blood flow. They also discussed the potential benefits of V1RAs in reducing these deleterious effects and improving neurological outcomes after stroke. They highlighted the results of animal studies and clinical trials that showed that V1RAs can decrease the infarct size, attenuate brain damage, and enhance the recovery of motor and cognitive functions. The authors suggested that V1RAs can be used as an adjunctive therapy for stroke, especially in combination with thrombolysis or mechanical thrombectomy.

The third paper discusses the challenges and opportunities of DBS for the treatment of PD and AD, and highlights the need for personalized and adaptive stimulation paradigms [79]. The authors reviewed the current state of the art and the future directions of DBS for these neurodegenerative disorders, focusing on the selection of optimal stimulation targets, parameters, and patterns. They emphasized the importance of tailoring the stimulation to the individual patient’s symptoms, disease stage, and neural activity, as well as adjusting the stimulation in real time based on the feedback from biomarkers, such as EEG, LFPs, or neurochemicals. They also explored the potential of DBS to modulate the neural circuits and networks involved in motor, cognitive, and emotional functions, and to restore the balance between excitation and inhibition in the brain.

The fourth paper evaluates the safety of a special waveform of transcranial electrical stimulation (TES) in vivo, and demonstrates that it can modulate cortical excitability without inducing seizures or neuronal damage [80]. The authors used a novel TES waveform, called the alternating current square wave (ACSW), which consists of alternating positive and negative pulses with a fixed duration and amplitude. They applied ACSW to the rat somatosensory cortex and measured the changes in cortical excitability, seizure susceptibility, and histological alterations. They found that ACSW increased the cortical excitability, as measured by the amplitude of the somatosensory evoked potentials (SEPs), but did not induce seizures, even at high intensities. Furthermore, ACSW did not cause any neuronal damage, inflammation, or apoptosis in the stimulated cortex. The authors concluded that ACSW is a safe and effective TES waveform for modulating cortical excitability (Table 1).

### 2.2. Neurodegenerative and Neuroinflammatory Disorders

#### 2.2.1. Alzheimer’s Disease and Related Disorders

AD is the most common cause of dementia, a progressive and irreversible decline in cognitive functions such as memory, language, reasoning, and judgment [112,113,114,115]. AD is characterized by the accumulation of amyloid plaques and neurofibrillary tangles in the brain, leading to neuronal loss and synaptic dysfunction [116,117,118]. Other forms of dementia include vascular dementia, caused by impaired blood flow to the brain; frontotemporal dementia, caused by degeneration of the frontal and temporal lobes; and Lewy body dementia, resulting from abnormal deposits of alpha-synuclein protein in the brain [119,120,121,122]. In this subsection, we review four papers that investigate the etiology, pathogenesis, biomarkers, and therapeutic strategies for AD and related disorders.

The first paper explores the potential role of the liver–brain axis in AD and aging, and how it is influenced by sex, isolation, and obesity [81]. The authors used a mouse model of AD and wildtype counterparts with normal aging where males exhibit obesity. They found that hepatic oxi-inflammation was associated with worse cognitive and behavioral impairments in the mice, and that this effect was more pronounced in male mice and in isolated and obese mice. They also observed that hepatic oxi-inflammation increased the brain AD–neuropathological levels and reduced the expression of neurotrophic factors and synaptic proteins. The authors suggested that hepatic oxi-inflammation and neophobia, a fear of novelty, could be potential targets for preventing or delaying AD and aging.

The second paper examines the risk factors and mechanisms for psychotic symptoms in AD, such as hallucinations, delusions, and agitation, using electronic medical records and deep learning models [82]. The authors analyzed the data of over 300,000 patients with AD, and identified several clinical and demographic variables that were associated with psychotic symptoms, such as age, sex, race, comorbidities, medications, and cognitive and functional status. They also used a convolutional neural network to extract features from the text of the medical records, which improved the prediction of psychotic symptoms. The authors concluded that electronic medical records and deep learning models could provide valuable insights into the etiology and management of psychotic symptoms in AD.

The third paper investigates the role of stromal interaction molecule (STIM) 1 and STIM2, two calcium sensors that regulate intracellular calcium homeostasis, in the pathophysiology of AD, using a mouse model of the disease [83]. The authors measured the expression and localization of STIM1 and STIM2 in the hippocampus of the mice, and found that both were reduced and in different places in the neurons of the Alzheimer’s mice compared to the control mice. Electrophysiological recordings and calcium imaging revealed that the Alzheimer’s mice exhibited impaired synaptic transmission and calcium signaling in the hippocampal neurons. Overexpressing STIM1 or STIM2 in the hippocampus of the Alzheimer’s mice improved their synaptic function and memory performance. The authors suggested that STIM1 and STIM2 could be novel targets for restoring calcium homeostasis and synaptic plasticity in AD.

The fourth paper evaluates the effect of intraoperative hypothermia, a common complication during surgery, on the vascular function and integrity of the rat hippocampus, a brain region that is vulnerable to ischemia and AD [84]. The authors induced hypothermia in the rats by lowering their body temperature to 28 °C for 2 h during surgery, and measured changes in the blood–brain barrier permeability, cerebral blood flow, and vascular reactivity in the hippocampus. They found that hypothermia increased the blood–brain barrier permeability and reduced the cerebral blood flow and vascular reactivity in the CA1 region of the hippocampus; these effects persisted for 24 h post surgery. Additionally, they observed that hypothermia impaired the spatial learning and memory in the rats, and that these deficits correlated with vascular dysfunction. The authors concluded that intraoperative hypothermia could induce vascular dysfunction and cognitive impairment in the hippocampus, potentially influencing the development and progression of AD (Table 1).

#### 2.2.2. Neural Regeneration and Repair

The nervous system is composed of billions of neurons and glial cells that communicate and cooperate to perform various functions, such as sensation, movement, cognition, and emotion [123,124,125,126,127]. However, the nervous system is also vulnerable to various forms of injury and disease, such as trauma, stroke, infection, degeneration, and malformation, which can impair or destroy neural functions and structures [128,129,130]. Consequently, there is a significant need to develop effective methods to enhance the recovery and restoration of neural function after injury or disease, such as stem cell therapy, gene therapy, tissue engineering, and neurotrophic factors [131,132,133,134,135]. In this subsection, we review three papers that explore the use of different methods for neural regeneration and repair.

The first paper reviews the techniques, mechanisms, potential applications, and challenges of somatic cell reprogramming for nervous system diseases [85]. Somatic cell reprogramming is a process that converts a differentiated cell into another cell type, such as a pluripotent stem cell or a neural cell, by introducing specific factors or stimuli. The authors summarize the current state of the art and the future directions of somatic cell reprogramming for generating neural cells, such as neurons, astrocytes, oligodendrocytes, and microglia, and for modeling and treating various nervous system diseases, such as AD, PD, spinal cord injury, and brain tumors. They also discuss the advantages and limitations of somatic cell reprogramming, including the efficiency, safety, scalability, and ethical issues, and propose possible solutions and strategies.

The second paper evaluates the effect of the autologous genetically enriched leucoconcentrate (AGEL) on the lumbar spinal cord morpho-functional recovery in a mini pig with thoracic spine contusion injury [86]. AGEL is a cell-based therapy consisting of autologous leukocytes that are genetically modified to overexpress the neurotrophin-3 gene, a potent factor promoting neural survival, differentiation, and regeneration. The authors induced a thoracic spine contusion injury in a mini pig, and injected AGEL into the lumbar spinal cord at 24 h and 7 days after injury. They measured changes in spinal cord morphology, electrophysiology, and locomotor function at different time points after injury and treatment. They found that AGEL significantly improved the spinal cord morphology, such as reducing the cavity size, increasing the tissue sparing, and enhancing the axonal growth and myelination. They also found that AGEL significantly improved the spinal cord electrophysiology, such as by increasing the amplitude and decreasing the latency of SEPs and motor-evoked potentials. Furthermore, AGEL significantly enhanced locomotor function, increasing Basso–Beattie–Bresnahan and grid walk scores. The authors concluded that AGEL is a promising therapy for spinal cord injury, and that the mini pig is a suitable animal model for preclinical studies.

The third paper investigates the role of the extracellular-signal-regulated kinase (ERK)1/2 signaling pathway in regulating the tubulin-binding cofactor B (TBCB) expression and affecting the astrocyte process formation after acute fetal alcohol exposure [87]. ERK1/2 is a key kinase that mediates various cellular processes, such as proliferation, differentiation, and survival. TBCB is a protein that regulates microtubule dynamics and stability, which are essential for the cytoskeleton and morphology of cells. The authors exposed rat fetuses to ethanol on the 12th day of gestation, and isolated the astrocytes from the cortex and hippocampus of the pups on postnatal day 1. They measured changes in the ERK1/2 activation, TBCB expression, and astrocyte process formation in vitro. They found that ethanol exposure decreased the ERK1/2 activation and TBCB expression, and impaired the astrocyte process formation in both regions. They also found that pharmacological inhibition or the genetic knockdown of ERK1/2 mimicked the effects of ethanol exposure, while pharmacological activation or the genetic overexpression of ERK1/2 reversed the effects of ethanol exposure. The authors suggested that the ERK1/2 signaling pathway regulates TBCB expression and affects astrocyte process formation after acute fetal alcohol exposure, and that this pathway could be a potential target for preventing or treating fetal alcohol spectrum disorders (Table 1).

#### 2.2.3. Rare and Genetic Neurological Disorders

The paper by Sivananthan et al. introduces the Buffy Coat Score (BCS) as a novel biomarker for assessing treatment response in neuronal ceroid lipofuscinosis type 2 (NCL2) [88]. The BCS is a quantitative measure derived from blood samples, specifically the buffy coat layer, which can reflect the cellular changes in response to therapy. This advancement is significant as it provides a less invasive, cost-effective, and timely method for monitoring disease progression and therapeutic efficacy in NCL2 patients, potentially improving patient management and treatment outcomes (Table 1).

### 2.3. Neuropsychiatric Disorders and Treatments

These papers explore the diagnosis, pathophysiology, and pharmacological or non-pharmacological interventions for various mental health conditions, such as depression, bipolar disorder (BD), anxiety, SCZ, and substance abuse.

#### 2.3.1. Depression and Antidepressants

Depression is a serious mood disorder that affects millions of people worldwide. It is characterized by persistent feelings of sadness, hopelessness, and loss of interest in daily activities, as well as physical symptoms such as fatigue, insomnia, and appetite changes [136,137,138]. Depression can impair the quality of life and increase the risk of suicide and other health problems [139,140,141,142]. The causes of depression are complex and multifactorial, involving genetic, biological, psychological, and environmental factors [143,144,145,146,147]. Treatment usually involves a combination of psychotherapy and pharmacotherapy, with antidepressants being the most commonly prescribed drugs [148,149,150,151]. In this subsection, we review two papers that explore the diagnosis, pathophysiology, and pharmacological interventions for major depressive disorder.

The first paper by Vasiliu provides a narrative review of the efficacy, tolerability, and safety of toludesvenlafaxine, a novel antidepressant that belongs to the class of serotonin and norepinephrine reuptake inhibitors (SNRIs) [89]. The author summarizes the results of preclinical and clinical studies that evaluated the pharmacokinetics, pharmacodynamics, and therapeutic effects of toludesvenlafaxine in comparison with other SNRIs, such as venlafaxine, duloxetine, and desvenlafaxine. The author also discusses the potential advantages and disadvantages of toludesvenlafaxine, such as its lower risk of drug interactions, its longer half-life, and its higher incidence of adverse events. The author concludes that toludesvenlafaxine is a promising antidepressant that may offer some benefits over existing SNRIs; however, more studies are needed to confirm its efficacy and safety in different populations and settings.

The second paper by Kalkman proposes a novel in vitro screen to detect new antidepressant principles based on the inhibition of microglial glycogen synthase kinase-3 beta (GSK3β) activity [90]. The author explains that microglia, the immune cells of the brain, play a key role in neuroinflammation, which is implicated in the pathogenesis of depression. GSK3β is a kinase that regulates various cellular processes, such as metabolism, proliferation, and apoptosis; its inhibition has antidepressant-like effects in animal models. The author suggests that different kinds of antidepressants, such as selective serotonin reuptake inhibitors, SNRIs, monoamine oxidase inhibitors, and ketamine, share a common mechanism of action: the inhibition of microglial GSK3β activity. The author proposes a simple and rapid assay that measures the GSK3β activity in microglial cells exposed to different compounds, which could be used to screen for novel antidepressant principles. This assay is hoped to facilitate the discovery and development of new and more effective antidepressants.

#### 2.3.2. Neural Oscillations and Cognitive Functions

Neural oscillations are rhythmic patterns of electrical activity that occur in the brain, and reflect the synchronization of neural populations [152,153,154,155]. Neural oscillations can be measured by various techniques, such as EEG or magnetoencephalography (MEG), and can be classified into different frequency bands, such as delta, theta, alpha, beta, and gamma [156,157,158,159]. Neural oscillations are involved in various cognitive functions, such as memory, attention, and decision making, and are modulated by various factors, such as sensory input, task demands, and emotional states [160,161,162,163]. Neural oscillations are also affected by neuropsychiatric disorders, including SCZ, BD, and autism, and may serve as biomarkers or targets for diagnosis and intervention [164,165,166,167]. In this subsection, we review two papers that explore the role of alpha oscillations, a type of brain wave that occurs in the range of 8–12 Hz, in various cognitive functions, and how they are affected by neuropsychiatric disorders.

The first paper provides a comprehensive review of the role of alpha oscillations among key neuropsychiatric disorders in the adult and developing human brain, based on evidence from the last 10 years of research [91]. The authors summarize the findings from EEG, MEG, and fMRI studies that investigated the alpha oscillations in SCZ, BD, major depressive disorder, anxiety disorders, obsessive compulsive disorder, post-traumatic stress disorder, attention-deficit/hyperactivity disorder, autism spectrum disorder, and Tourette syndrome. The authors discuss the similarities and differences in alpha oscillations across these disorders, and how they relate to the clinical symptoms, cognitive impairments, and neurodevelopmental trajectories. The authors also highlight the potential applications of alpha oscillations for the diagnosis, prognosis, and treatment of these disorders, and suggest some future directions and challenges for the field.

The second paper examines the cost of imagined actions in a reward-valuation task, and how it is modulated by alpha oscillations. The authors conducted an EEG experiment in which healthy participants had to choose between two options that differed in the amount of reward and the number of actions required to obtain it [92]. Participants had to either perform or imagine the actions, and the authors measured the alpha oscillations during the choice and the action phases. The authors found that the participants preferred the option with fewer actions, regardless of the reward amount, and that this preference was stronger when they had to imagine the actions rather perform them. The authors also found that alpha oscillations increased during the choice phase, and that this increase correlated with the preference for fewer actions. The authors suggested that alpha oscillations reflect the inhibition of irrelevant or costly actions, and that imagined actions have a higher cost than performed actions (Table 1).

#### 2.3.3. Neurotrophic Factors and Genetic Variants

Neurotrophic factors are molecules that support the survival, growth, and differentiation of neurons and glial cells in the nervous system. They also regulate various aspects of neural function, such as synaptic transmission, plasticity, and neurogenesis [168,169,170,171]. One of the most widely studied neurotrophic factors is nerve growth factor (NGF), which binds to its receptor, nerve growth factor receptor (NGFR), also known as p75NTR [172,173,174]. NGF and NGFR play crucial roles in the development and maintenance of the cholinergic system, which is important for learning, memory, and cognition [163,175,176,177]. They are also implicated in the pathophysiology of various neuropsychiatric disorders, such as SCZ, BD, and AD, which are characterized by cognitive impairment, neuroinflammation, and neurodegeneration [161,178,179,180]. In this subsection, we review one paper that investigates the role of the NGFR gene and its single-nucleotide polymorphisms (SNPs), rs2072446 and rs11466162, in psychiatric disorders.

The paper investigates the role of the NGFR gene and its SNPs, rs2072446 and rs11466162, in psychiatric disorders, such as SCZ, BD, and AD [93]. The authors performed a meta-analysis of 18 studies that examined the association between these SNPs and the risk or severity of these disorders. The authors found that the NGFR gene and its SNPs were significantly associated with psychiatric disorders, especially SCZ and BD. The authors also found that the NGFR gene and its SNPs interacted with other genes and environmental factors to influence the pathogenesis and progression of these disorders. The authors suggested that the NGFR gene and its SNPs could be potential biomarkers or therapeutic targets for psychiatric disorders.

#### 2.3.4. Neurotransmission and Neuroprotection

Neurotransmission is the process of communication between neurons and other cells in the nervous system, mediated by chemical messengers called neurotransmitters. Neurotransmitters can modulate the neural activity and synaptic plasticity, which are essential for learning, memory, and cognition [160,162,181,182]. Neurotransmitters can also confer neuroprotection, which is the ability of the nervous system to resist or recover from damage caused by various insults, such as oxidative stress, excitotoxicity, inflammation, and degeneration [183,184,185,186,187]. One of the most important neurotransmitters in the nervous system is glutamate, which is the main excitatory neurotransmitter that mediates rapid synaptic transmission and long-term potentiation [188]. However, excessive glutamate can also cause excitotoxicity, the overstimulation and subsequent death of neurons [189]. Therefore, maintaining a balance between glutamate and its antagonists, such as kynurenic acid, is crucial for neural function and integrity [190]. In this subsection, we review one paper that explores the role of kynurenic acid (KYNA) in neurotransmission and neuroprotection.

The paper investigates the effect of KYNA on memory enhancement and its mechanisms in neurotransmission [94]. KYNA is an endogenous metabolite of tryptophan that acts as an antagonist of glutamate receptors, such as N-methyl-D-aspartate, α-amino-3-hydroxy-5-methyl-4-isoxazolepropionic acid, and kainate receptors. The authors administered KYNA to rats and mice and measured changes in their memory performance, synaptic transmission, and neurotransmitter levels. The authors found that KYNA improved the memory performance of the animals in various tasks, such as passive avoidance, object recognition, and the Morris water maze. KYNA also increased synaptic transmission and the levels of acetylcholine, dopamine, and serotonin in the hippocampus and the cortex, which are brain regions involved in memory formation and consolidation. The authors suggest that KYNA enhances memory by modulating the glutamatergic and cholinergic systems, and increasing the levels of other neurotransmitters involved in memory and cognition. They also propose that KYNA may have neuroprotective effects against oxidative stress and neuroinflammation, which are associated with neurodegenerative diseases such as AD (Table 1).

#### 2.3.5. Neuromodulation and Neuroregeneration

Neuromodulation is the process of altering neural activity and function by applying external stimuli such as light, electric currents, magnetic fields, or drugs to specific brain regions or networks [14,191]. Neuroregeneration involves restoring neural structures and functions by promoting the survival, growth, and differentiation of neurons and glial cells [192,193]. Both processes are important for enhancing the recovery and restoration of neural function after injury or disease, such as trauma, stroke, infection, degeneration, and malformation [194]. In this subsection, we review two papers that explore the use of different methods for neuromodulation and neuroregeneration.

The first paper investigates the effect of heterologous fibrin biopolymer and photobiomodulation on morphofunctional improvements of the facial nerve and muscles after injury [95]. Heterologous fibrin biopolymer, a biomaterial derived from horse blood, acts as a scaffold and hemostatic agent for tissue repair. Photobiomodulation, a technique using low-level laser therapy, modulates cellular metabolism and function. The authors induced a facial nerve injury in rats and treated them with heterologous fibrin biopolymer, photobiomodulation, or both. They measured the changes in facial nerve morphology, electrophysiology, and functionality, as well as facial muscle histology at different time points after injury and treatment. They found that the combination of heterologous fibrin biopolymer and photobiomodulation significantly improved recovery of the facial nerve and muscles compared to the control or the single treatments. This combination treatment also increased the expression of neurotrophic factors, such as NGF and brain-derived neurotrophic factor, which are crucial for neural survival, differentiation, and regeneration. The authors concluded that heterologous fibrin biopolymer and photobiomodulation are effective methods for enhancing neuromodulation and neuroregeneration of the facial nerve and muscles after injury.

The second paper examines the establishment of a mouse model of recurrent primary dysmenorrhea, a common gynecological disorder characterized by painful menstrual cramps [96]. The authors used a chemical agent, zymosan, to induce an inflammatory response in the uterus of female mice. They measured changes in uterine contraction, blood flow, and pain behavior, as well as the expression of inflammatory mediators such as prostaglandins and cytokines. They found that zymosan injection caused significant increases in uterine contraction, blood flow, and pain behavior, along with the expression of inflammatory mediators in the mice. These effects were repeated in the subsequent estrous cycles, mimicking the clinical features of recurrent primary dysmenorrhea. The authors suggested that this mouse model could be used to study the pathophysiology and pharmacology of recurrent primary dysmenorrhea and to test the efficacy of potential treatments, such as neuromodulators and neuroprotective agents (Table 1).

## 3. Conclusions

The topic “Emerging Translational Research in Neurological and Psychiatric Diseases: From In Vitro to In Vivo Models, from Animals to Humans, from Qualitative to Quantitative Methods 2.0” represents a significant advancement in our quest to understand and treat neurological and psychiatric disorders. The 22 papers in this collection span a broad spectrum of research, from the molecular bases of neurodegenerative diseases to innovative therapeutic approaches for neuropsychiatric conditions. They reflect the collaborative efforts of scientists and clinicians who are committed to unraveling the complexities of the brain and mind. The insights gleaned from these studies underscore the importance of multidisciplinary but integrative approaches, integrating neuroimaging, neurostimulation, genetic analysis, and computational modeling [195,196,197,198,199,200]. As we synthesize knowledge from in vitro experiments, animal models, and human clinical trials, we pave the way for transformative breakthroughs that can be translated into effective interventions. This collection not only highlights the current achievements in the field, but also illuminates the path forward. It encourages ongoing dialogue and research collaboration, fostering an environment where scientific curiosity and clinical need converge to inspire innovation. As we continue to push the boundaries of our understanding, we remain hopeful that the work encapsulated in these pages will lead to improved outcomes for individuals affected by neurological and psychiatric diseases worldwide.

## Figures and Tables

**Table 1:** Major subtopics covering the Topic “Emerging Translational Research in Neurological and Psychiatric Diseases”.

Subtopics		Ref.
1. Neuroimaging and Neurostimulation Techniques		
	a. Neuroimaging Techniques for Brain Morphometry and Function	[75,76]
	b. Neurostimulation Techniques for Brain Modulation and Therapy	[77,78,79,80]
2. Neurodegenerative and Neuroinflammatory Disorders		
	a. Alzheimer’s Disease and Related Disorders	[81,82,83,84]
	b. Neural Regeneration and Repair	[85,86,87]
	c. Rare and Genetic Neurological Disorders	[88]
3. Neuropsychiatric Disorders and Treatments		
	a. Depression and Antidepressants	[89,90]
	b. Neural Oscillations and Cognitive Functions	[91,92]
	c. Neurotrophic Factors and Genetic Variants	[93]
	d. Neurotransmission and Neuroprotection	[94]
	e. Neuromodulation and Neuroregeneration	[95,96]

## Data Availability

Data sharing is not applicable to this article.

## Abbreviations

AD	Alzheimer’s disease
ACSW	Alternating current square wave
AGEL	Autologous genetically enriched leucoconcentrate
AVP	Arginine–vasopressin
BCS	Buffy Coat Score
BOLD	Blood-oxygen-level-dependent
BD	Bipolar disorder
DBS	Deep brain stimulation
EEG	Electroencephalography
ERK	Extracellular-signal-regulated kinase
fMRI	Functional magnetic resonance imaging
GSK3β	Glycogen synthase kinase-3 beta
KYNA	Kynurenic acid
LFPs	Local field potentials
NGF	Nerve growth factor
NGFR	Nerve growth factor receptor
MEG	Magnetoencephalography
MRI	Magnetic resonance imaging
NCL2	Neuronal ceroid lipofuscinosis type 2
PD	Parkinson’s disease
SCZ	Schizophrenia
SEPs	Somatosensory evoked potentials
SNPs	Single-nucleotide polymorphisms
SNRIs	Serotonin and norepinephrine reuptake inhibitors
STIM	Stromal interaction molecule
TBCB	Tubulin-binding cofactor B
TMS	Transcranial magnetic stimulation
TES	Transcranial electrical stimulation
V1RAs	Vasopressin type 1 receptor antagonists
